# Burn Injury Triggered Dysfunction in Dendritic Cell Response to TLR9 Activation and Resulted in Skewed T Cell Functions

**DOI:** 10.1371/journal.pone.0050238

**Published:** 2012-11-26

**Authors:** Haitao Shen, Patricia E. de Almeida, Kyung H. Kang, Pamela Yao, Camie W. Chan

**Affiliations:** 1 Department of Cell Biology and Human Anatomy, University of California Davis, Davis, California, United States of America; 2 Institute for Pediatric Regenerative Medicine, Shriners Hospitals for Children Northern California, Sacramento, California, United States of America; 3 Laboratory of Pathology, Hebei Medical University, Shijiazhuang, China; 4 Stem Cell & Regenerative Medicine Consortium, The University of Hong Kong, Pokfulam, Hong Kong; 5 Department of Anatomy, The University of Hong Kong, Pokfulam, Hong Kong; 6 Department of Medicine, The University of Hong Kong, Pokfulam, Hong Kong; Carl-Gustav Carus Technical University-Dresden, Germany

## Abstract

Severe trauma such as burn injury is often associated with a systemic inflammatory syndrome characterized by a hyperactive innate immune response and suppressed adaptive immune function. Dendritic cells (DCs), which sense pathogens via their Toll-like receptors (TLRs), play a pivotal role in protecting the host against infections. The effect of burn injury on TLR-mediated DC function is a debated topic and the mechanism controlling the purported immunosuppressive response remains to be elucidated. Here we examined the effects of burn injury on splenic conventional DC (cDC) and plasmacytoid DC (pDC) responses to TLR9 activation. We demonstrate that, following burn trauma, splenic cDCs’ cytokine production profile in response to TLR9 activation became anti-inflammatory dominant, with high production of IL-10 (>50% increase) and low production of IL-6, TNF-α and IL-12p70 (∼25–60% reduction). CD4+ T cells activated by these cDCs were defective in producing Th1 and Th17 cytokines. Furthermore, burn injury had a more accentuated effect on pDCs than on cDCs. Following TLR9 activation, pDCs displayed an immature phenotype with an impaired ability to secrete pro-inflammatory cytokines (IFN-α, IL-6 and TNF-α) and to activate T cell proliferation. Moreover, cDCs and pDCs from burn-injured mice had low transcript levels of TLR9 and several key molecules of the TLR signaling pathway. Although hyperactive innate immune response has been associated with severe injury, our data show to the contrary that DCs, as a key player in the innate immune system, had impaired TLR9 reactivity, an anti-inflammatory phenotype, and a dysfunctional T cell-priming ability. We conclude that burn injury induced impairments in DC immunobiology resulting in suppression of adaptive immune response. Targeted DC immunotherapies to promote their ability in triggering T cell immunity may represent a strategy to improve immune defenses against infection following burn injury.

## Introduction

Burns are a serious global health problem, according to the World Health Organization, with over 195,000 related deaths each year. Burn injury alters host immune functions, predisposing patients to opportunistic and nosocomial infections, sepsis, and multiple organ system dysfunction and failure. Burn injury often leads to a systemic inflammatory state, which has been attributed to the resulting exacerbated innate immune response, referred to as systemic inflammatory response syndrome (SIRS) [1], [2]. Macrophages, which upregulate Toll-like receptor 4 (TLR4) responses, are believed to be the major source of inflammatory mediators during SIRS [3], [4], [5], [6]. The adaptive immune system, by contrast, acquires a suppressive phenotype characterized by a reduced T helper (Th) 1 and cytotoxic T cell response, and heightened T regulatory (Treg) cell activity [7], [8], [9], [10], [11], [12]. The mechanism responsible for initiating and controlling this immunosuppressive response after burn injury remains to be elucidated. Dendritic cells (DCs) are known to play a key role in linking the innate and adaptive arms of the immune system [13]. The heterogeneous DC family is mainly classified into conventional and plasmacytoid DCs. Conventional DCs (cDCs) efficiently induce antigen-specific T-cell responses [13], whereas plasmacytoid DCs (pDCs) produce high amounts of type I interferon (IFN) [14]. Recent studies have demonstrated that pDCs play an important role in activating cDCs [15], [16], NK cells, B cells, and T cells [17], [18]. In addition, we and others have reported an immune cell population named interferon-producing killer DCs (IKDCs) which control infection by possessing an unique ability of directly lysing infected cells followed by presenting the Ags to T cells [19], [20], [21]. Given that these DC subsets have unique functional characteristics, we have, in this study, compared the effects of severe injury on the different DC subpopulations.

**Figure 1:**
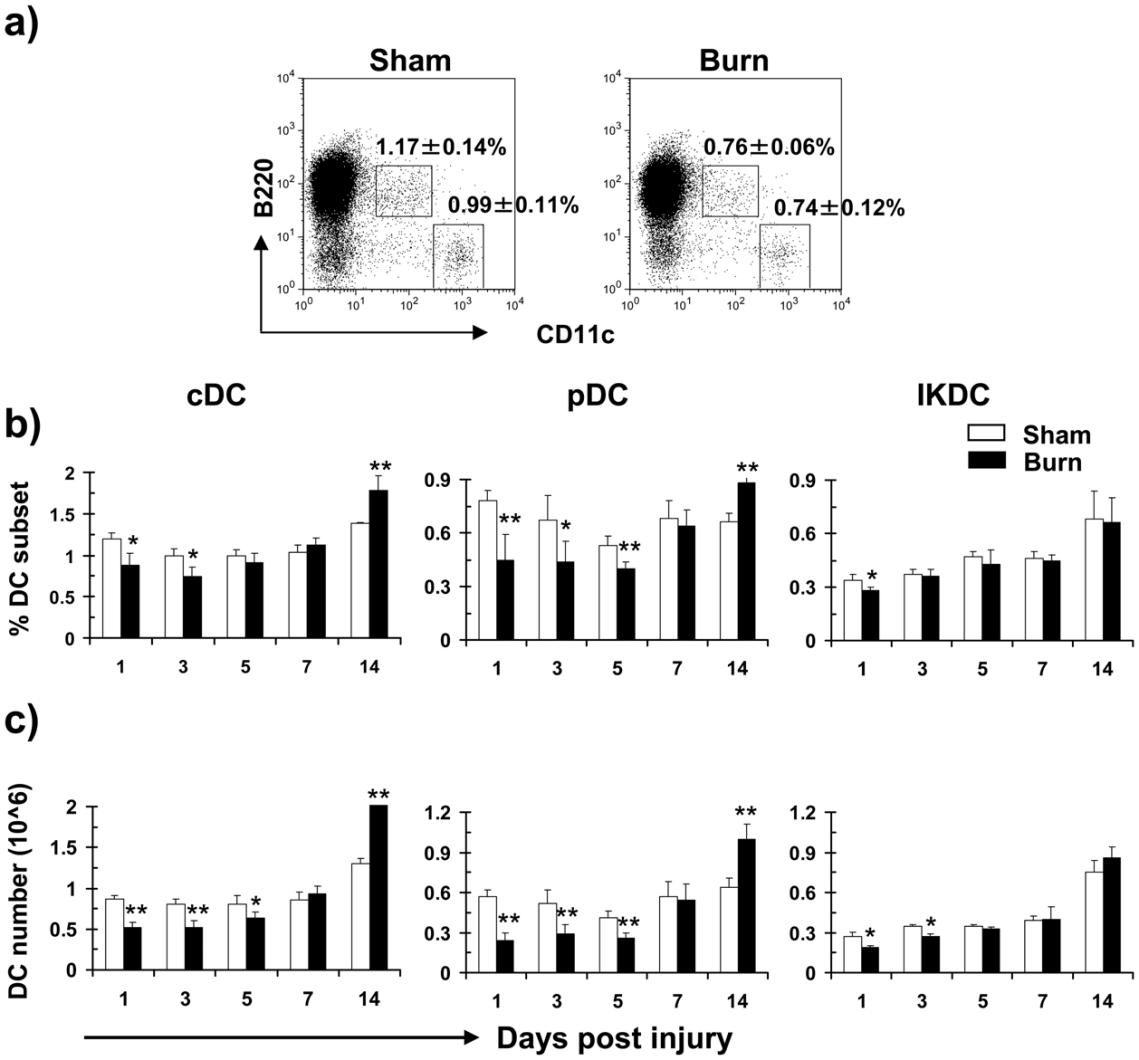
Reduced numbers and percentages of splenic cDCs and pDCs following burn injury. Mice were subjected to non-lethal thermal injury and total splenocytes were purified and stained using antibodies for distinct surface markers. The effect of burn injury on each DC subset was examined. (a) A representative FACS plot demonstrating the percentages of splenic cDCs (CD11c^hi^B220^neg^) and CD11c^low^B220^+^ DC subpopulations at d3 post-injury in comparison to sham. The CD11c^low^B220^+^ population comprises of pDCs (CD11c^low^B220^+^PDCA1^+^) and IKDCs (CD11c^low^B220^+^DX5^+^). Percentages of CD11c^hi^B220^neg^ cDCs and CD11c^low^B220^+^ DCs are shown as mean ± SEM (n = 9, 3 independent experiments). Percentages (b) and absolute numbers (c) of splenic cDCs, pDCs and IKDCs at various time points post burn and sham injury are shown. Data are shown as mean ± SEM (n = 9, 3 independent experiments). *P<0.05; **p<0.01, sham versus burn by ANOVA.

Efficient priming of T cells relies on appropriate DC maturation, which is elicited in response to recognition of specific pathogen-associated molecular patterns (PAMPs) via pattern recognition receptors (PRRs), including toll-like receptors (TLRs) [22]. To date, ten functional TLRs have been identified in humans; each recognizes unique PAMPs to induce immune activation. For example, in response to Gram-negative bacteria infection, TLR4 plays an important role in triggering effective host immunity by recognizing endotoxin lipopolysaccharides (LPS), integral components of the outer bacterial membrane [23]. TLR9 importantly controls bacterial and viral infections, such as those of *Escherichia coli, Pseudomonas aeruginosa*, and DNA viruses, by recognizing unmethylated CpG DNA motifs [24], [25], [26]. Recent findings report that activation of multiple TLRs is required for a complete immune response during microbial challenge [25], [27], [28]. Burn injury patients are susceptible to infection from a variety of pathogens, including Gram-positive and Gram-negative bacteria as well as viruses. Here, we examined the effect of burn injury on DC responses to TLR9 activation. Our data suggest that DCs do not contribute to post-injury SIRS; rather, DCs function is skewed toward an immunosuppressive phenotype, with an impaired ability to activate T cell responses.

**Table 1:** Reduced numbers and percentages of splenic CD4^+^CD8^−^ and CD4^−^CD8^+^ cDCs following burn injury.^1^

		Cell (%)		Cell number (10^6^)	
	Days post-injury	Sham	Burn	Sham	Burn
CD4^+^CD8^−^cDCs	d1	0.75±0.04	0.56±0.09**	0.56±0.02	0.34±0.06*
	d3	0.70±0.04	0.53±0.06**	0.57±0.07	0.35±0.07*
	d5	0.64±0.04	0.59±0.06	0.50±0.06	0.38±0.06*
	d7	0.69±0.03	0.63±0.10	0.55±0.04	0.53±0.05
CD4^−^CD8^+^cDCs	d1	0.15±0.03	0.12±0.02*	0.10±0.02	0.07±0.01*
	d3	0.15±0.04	0.10±0.02*	0.11±0.03	0.07±0.01*
	d5	0.13±0.04	0.16±0.04	0.10±0.02	0.11±0.03
	d7	0.14±0.02	0.17±0.02*	0.11±0.03	0.14±0.02*
CD4^−^CD8^−^cDCs	d1	0.21±0.03	0.23±0.03	0.16±0.01	0.14±0.02
	d3	0.16±0.02	0.16±0.02	0.13±0.01	0.11±0.01
	d5	0.16±0.05	0.16±0.03	0.14±0.03	0.12±0.03
	d7	0.18±0.04	0.27±0.03*	0.14±0.04	0.23±0.06*

1 Data are shown as mean ± SEM (n = 9, 3 independent experiments).

* *P*<0.05, sham versus burn by ANOVA.

## Materials and Methods

### Mice

BALB/c (female, 6–8 weeks old) mice were purchased from The Jackson Laboratory (West Sacramento, CA). DO11.10 transgenic (Tg) mice expressing MHC class II-restricted TCR for ovalbumin (OVA) 323–339 (OVA_323–339_) peptide and Clone 4 Tg mice expressing MHC class I-restricted TCR for influenza virus A/PR/8 hemagglutinin (HA) (The Jackson Laboratory; Bar Harbor, Maine) were used to test antigen-specific T cell responses. This study was carried out in strict accordance with the recommendations in the Guide for the Care and Use of Laboratory Animals of the National Institutes of Health. The protocol was approved by the Committee on the Ethics of Animal Experiments of the University of California Davis (Permit Number: 12630).

**Figure 2:**
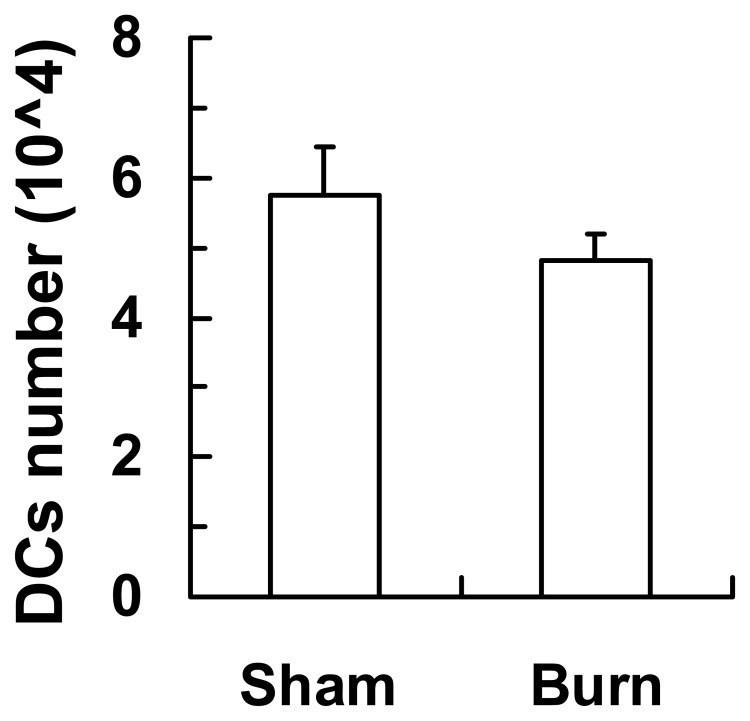
Absolute counts of infiltrating DCs recruited to the burn and sham injury site. Three days post burn injury, the skin specimens of burn and sham injury (2 cm×2 cm) were harvested and digested in Dispase II and collagenase D. Cells were isolated and stained for CD11c^+^ for DCs followed by flow cytometry analysis. Absolute numbers of infiltrating CD11c^+^ DCs are shown as mean ± SEM (n* = *8, 4 independent experiments).

### Reagents and Flow Cytometry

All fluorochrome-labeled mAbs were purchased from BD Biosciences (San Jose, CA), with the exception of PE-conjugated anti-mPDCA-1 (Miltenyi Biotec; CA). The following mAb staining profile was employed: anti-mouse CD3e (145-2C11), anti- mouse CD4 (L3T4), anti-CD8α (53-6.7), anti-CD11c (HL3), anti-B220/CD45R (RA3-6B2), anti-CD40 (3/23), anti-CD80 (1G10), anti-CD86 (GL1), anti-I-E^k/d^ (14-4–42). Prior to staining with labeled mAbs, FcγRII/III receptors were blocked with anti-CD16/32 (2.4G2) mAb. Flow cytometry analysis was performed using a Beckman-Coulter CyAn ADP (Fullerton, CA). CpG ODN1668 (TCCATGACGTTCCTGATGCT) and ODN2395 (TCGTCGTTTTCGGCGCGCGCCG) were purchased from Operon (Huntsville, AL), and OVA_323–339_ (Ile-Ser-Gln-Ala-Val-His-Ala-Ala-His-Ala-Glu-Ile-Asn-Glu-Ala-Gly-Arg) and hemagglutanin (HA) (lle-Tyr-Ser-Thr-Val-Ala-Ser-Ser-Leu) peptides from American Peptide Company (Sunnyvale, CA). Buprenorphrine was purchased from Reckitt Pharmaceuticals (Richmond, VA).

**Table 2:** Ratios of splenic DC subsets following burn injury.^1^

	Ratio		
	Days post-injury	sham	burn
CD4^+^CD8^−^cDCs:CD4^−^CD8^+^cDCs	D1	5.01±0.77	4.79±1.18
	D3	5.08±1.50	4.78±1.48
	D5	4.60±0.91	3.99±1.17
	D7	5.20±0.99	3.72±0.65*
cDCs:pDCs	D1	1.53±0.09	1.77±0.36
	D3	1.49±0.23	1.63±0.20
	D5	1.87±0.18	2.26±0.29*
	D7	1.52±0.26	1.79±0.29

1 Data are shown as mean ± SEM (n = 9, 3 independent experiments).

* *P*<0.05, sham versus burn by ANOVA.

### Burn Injury Model

To study the effect of burn injury on DCs, a thermal injury protocol was followed, as described previously [29]. Briefly, dorsa of female BALB/c mice (6–8 weeks old) were shaved one day prior to the experiment. Immediately prior to burn injury, mice were anesthetized with 2.5% isoflurane, and 25% of the dorsal surface was immersed in either 90°C (burn) or 24°C isothermic water (sham) for 9s. Buprenorphrine (0.03 mg/mouse) and 0.9% saline (1 ml) were given intraperitoneally for analgesia and fluid resuscitation immediately after injury.

**Table 3:** Cell numbers and percentages of wound draining lymph node cDCs and pDCs following burn injury.^1^

		Cells (%)		Cells number (10^4^)	
	Days post-injury	Sham	Burn	Sham	Burn
cDCs	d1	0.78±0.02	0.51±0.10*	5.70±1.69	5.32±0.96
	d3	0.73±0.18	0.58±0.10	6.08±2.86	4.52±1.25
	d5	0.65±0.14	0.51±0.10	6.80±0.11	5.56±1.12
pDCs	d1	0.66±0.04	0.22±0.03**	4.25±0.44	1.77±0.35*
	d3	0.63±0.07	0.52±0.03*	5.04±1.28	4.04±0.78
	d5	0.43±0.04	0.44±0.06	4.29±0.68	4.95±1.24

1 Data are shown as mean ± SEM (n = 9, 3 independent experiments).

** *P*<0.001;

* *p*<0.05, sham versus burn by ANOVA.

### Total Cell Isolation and DC Counts

Cells were isolated from spleens and wound draining lymph nodes (LNs) (i.e. two axillary, two brachial, three mesenteric and two inguinal) on days 1, 3, 5, and 7 post-burn and -sham injuries, as previously described [19]. Briefly, spleens were chopped into small fragments, subjected to digestion by Liberase (27 WU/ml; Roche, Indianapolis, IN) and DNase I (0.1%; Roche) at room temperature for 20 min, then treated with EDTA (100 mM). LNs were pooled and smashed without digestion. Total lymphocytes were collected by density centrifugation in Nycodenz medium (Accurate Chemical and Scientific Corporation, Westbury, NY). To determine the number of each DC subset upon burn injury, cells were stained with APC-conjugated anti-mouse CD11c, PE-Texas Red-conjugated anti-mouse B220, FITC-conjugated anti-mouse PDCA, and biotin-conjugated anti-mouse MHC Class-II. Percentages of DC subsets were analyzed by flow cytometry (i.e. cDCs as CD11c^high^B220^−^; pDCs as CD11c^low^B220^+^PDCA^+^; IKDCs as CD11c^low^B220^+^DX5^+^). To determine the number of DCs infiltrated to the site of burn injury, skin specimens (2 cm×2 cm) were isolated from burn- and sham-injured mice then chopped and digested in Dispase II (0.4 U/ml) for 2 hr, followed by collagenase D (1 mg/ml) treatment for 30 min at 37°C. Cells were collected and stained for anti-CD11c. Absolute DC numbers were determined based on the DC percentages quantified by flow cytometry and the total cell counts.

**Figure 3:**
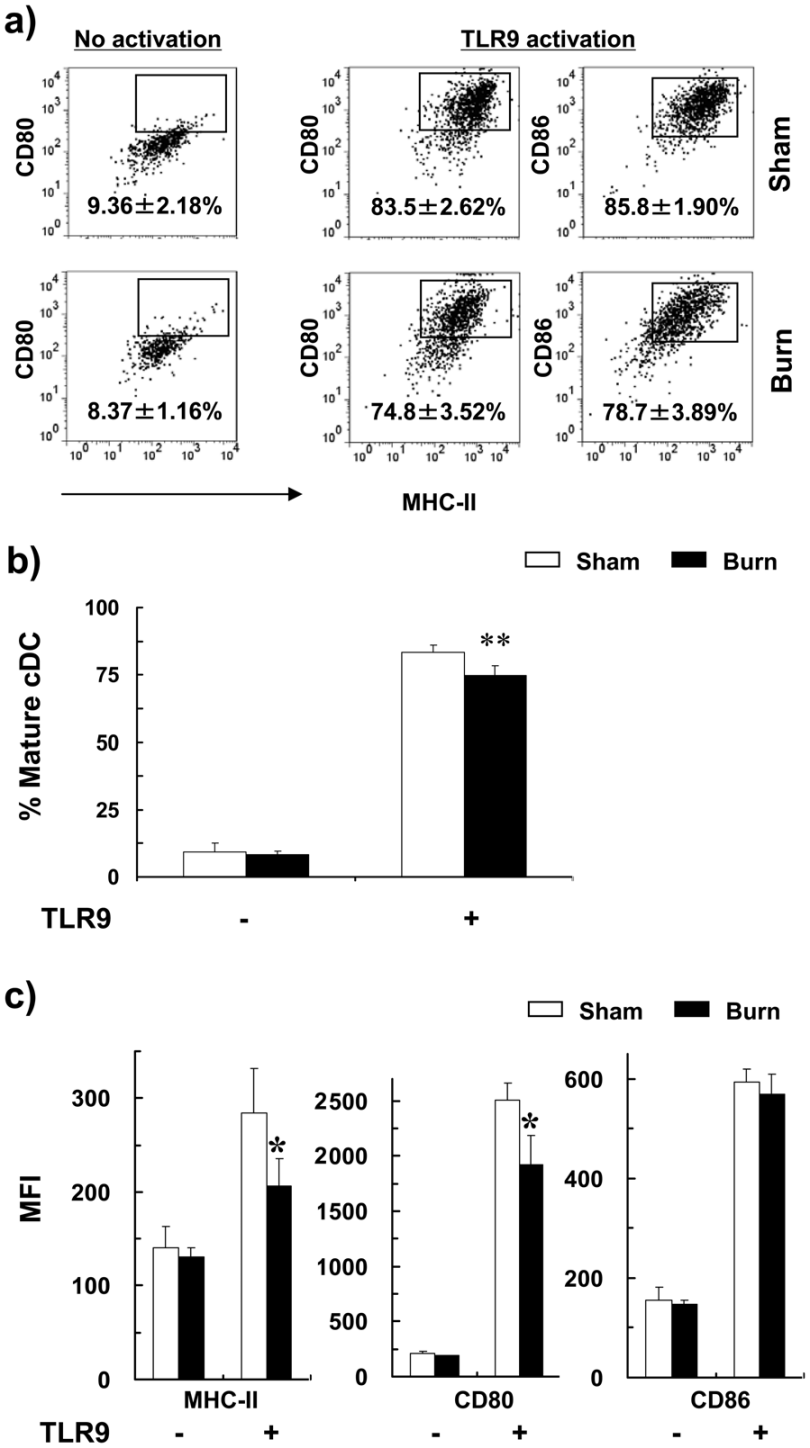
Burn injury impaired TLR9-induced cDC maturation. Three days post-injury, total splenic CD11c^+^ DCs were activated with or without CpG (24 hr) and stained for CD11c, B220, CD80, CD86 and MHC II. CDCs were gated as CD11c^hi^B220^−^ and the expression of CD80, CD86 and MHC II were analyzed by flow cytometry. (a) Representative FACS plots of non-activated (left) and TLR9-activated (right) cDCs of burn and sham mice. Percentages of mature cDCs (MHCII^hi^CD80^hi^CD86^hi^) are shown as mean ± SEM (n = 9, 3 independent experiments). (b) Percentages of MHCII^hi^CD80^hi^CD86^hi^ mature cDCs and (c) mean fluorescence intensity (MFI) of MHC-II, CD80 and CD86 expression on cDCs with and without TLR9 activation are shown as mean ± SEM (n = 9, 3 independent experiments). *P<0.05; **p<0.01, sham versus burn by ANOVA.

### DC Preparation and in vitro Challenge

Enriched DCs were prepared by depleting T and B cells from total splenocytes using purified rat anti-mouse CD3 and anti-mouse CD19 mAbs, then anti-rat IgG magnetic beads (Qiagen), followed by further enrichment using CD11c^+^ magnetic beads (CD11c^+^ isolation kit, Miltenyi Biotec). The enriched CD11c^+^ DCs were seeded in a 96-well round-bottom tissue culture plate (5×10^5^ cells/well) in RPMI-1640 supplemented with 10% FCS, L-glutamine, Na pyruvate, Hepes, non-essential amino acids and 10^−5^ M β-ME (Complete Medium) with GM-CSF (10 ng/ml; Peprotech, NJ) and challenged with the TLR9 agonist, unmethylated CpG ODN 1668 (CpG; 6 µg/ml). The effect of injury on maturation of cDCs was examined at 24 h post-TLR9 activation and pDCs at 40 h post-activation. After activation, cells were washed and stained with APC-conjugated anti-mouse CD11c, PE Texas Red-conjugated anti-mouse B220, FITC-conjugated anti-mouse PDCA, PE-conjugated anti-mouse CD80, or CD86 and biotin-conjugated MHC Class-II with streptavidin PE-Cy7 as secondary. cDCs and pDCs were analyzed as CD11c^hi^B220^−^ and CD11c^low^B220^+^PDCA^+^, respectively, by flow cytometry. In some cases, cDCs and pDCs were further prepared from the enriched DC population to a higher purity of >98% using a BD FACSAria II cell sorter (cDCs: CD11c^high^B220^−^DX5^−^PDCA^−^; pDCs: CD11c^low^B220^+^PDCA^+^). The sorted cDCs or pDCs were seeded in a 96-well U-bottom plate (2×10^5^ cells/well) and challenged with CpG (6 µg/ml). IL-3 (10 ng/ml; Peprotech) was added to the pDC culture to maintain viability. Supernatants were harvested 18–20 hr later and tested for levels of IL-6, IL-10, IFN-γ, TNF-α, and IL-12p70 using a BD Mouse Inflammation Cytometric Bead Array (CBA; BD Biosciences) following the manufacturer’s instructions. IL-12p70 (R&D Systems) and IFN-α (PBL Interferon Source, Piscataway, NJ) production was validated by ELISA assay.

**Figure 4:**
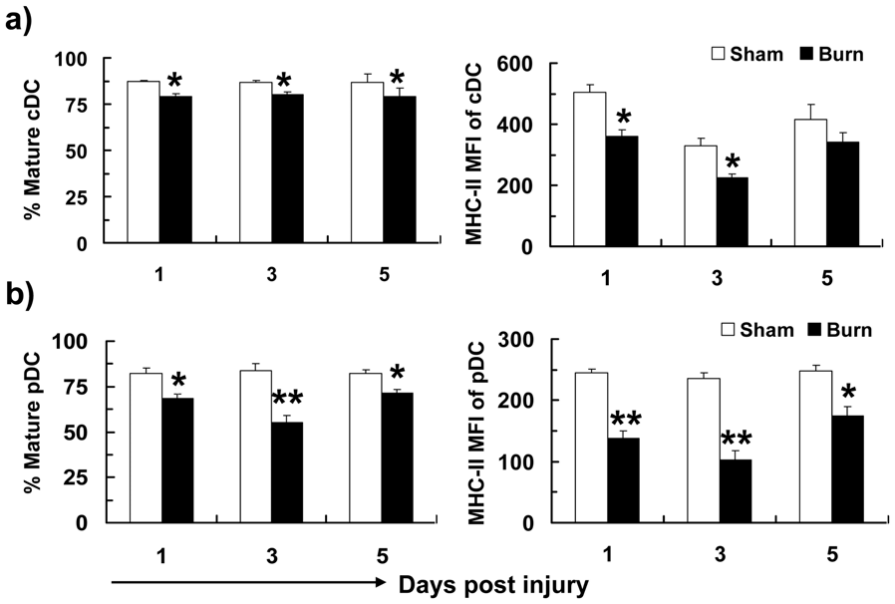
Reduced TLR9-mediated DC maturation following burn injury. Total splenic CD11c^+^ DCs were purified at different time point post-burn/sham injury and subjected to CpG activation. TLR-activated cells were stained for CD11c, B220, PDCA1, CD80, CD86 and MHC II expression and CDCs were gated as CD11c^hi^B220^−^ and pDCs as CD11c^low^B220^+^PDCA1^+^. Percentages of MHCII^hi^CD80^hi^CD86^hi^ mature cDCs (a) and pDCs (b), as well as MFI of MHC-II expression on cDCs (a) and pDCs (b) are shown as mean ± SEM (n >9, 3–5 independent experiments). *P<0.05, **p<0.01, sham versus burn by ANOVA.

### T Cell Proliferation Assay

OVA-specific CD4^+^ and HA-specific CD8^+^ transgenic T cells from DO11.10 and Clone-4 TCR Tg mice, respectively, were purified using CD4^+^ and CD8^+^ T cell isolation kits (Miltenyi) with a purity >95%. FACS-sorted cDCs and pDCs (2×10^4^ cells/well) were activated with CpG1668 (6 µg/ml) and pulsed with various concentrations of OVA_323–339_ or HA peptide (10 - 0.01 µg/ml) for 18–20 hr, then washed and co-cultured with CFSE-labelled CD4^+^ or CD8^+^ T cells (2×10^5^/well). Three days later, proliferation of T cells was assessed by flow cytometry, by CFSE dilution. Supernatant was collected to determine IL-2, IL-4, IL-6, IFN-γ, TNF-α, IL-17A, and IL-10 levels with a mouse Th1/Th2/Th17 CBA kit (BD Bioscience).

### RNA Isolation and qRT/PCR

Three days after burn injury, splenic cDCs and pDCs were purified to a purity of >98%, as described above, and were stored in TriZol (invitrogen) until further processing. RNA was isolated using a RNeasy Plus Mini Kit (QIAGEN, CA) and cDNA was prepared using a Reaction Ready First Strand cDNA Synthesis Kit (SuperArray Bioscience Corp., MD). The expression of genes related to TLR-mediated signal transduction was examined by real-time PCR Array using a SuperArray real-time PCR Kit (SuperArray Bioscience Corp., MD). Fold changes of transcript levels of genes in DCs of burn mice were calculated relative to those in the sham group. Transcript levels of TLR9 was evaluated by qPCR, performed using a SYBR@Green ER^TM^qPCR SuperMix Kit (Invitrogen) using the forward primer 5′-CGT TTC TCG GTG CTG GAC CTA AGC G-3′ and the reverse primer 5′-CTG AAA GGC ATT GGT GTG GTT G-3′. Gene expression was normalized to the housekeeping gene β-actin. Relative gene expression was calculated by the comparative ΔΔCt method, according to the manufacturer’s instruction.

**Figure 5:**
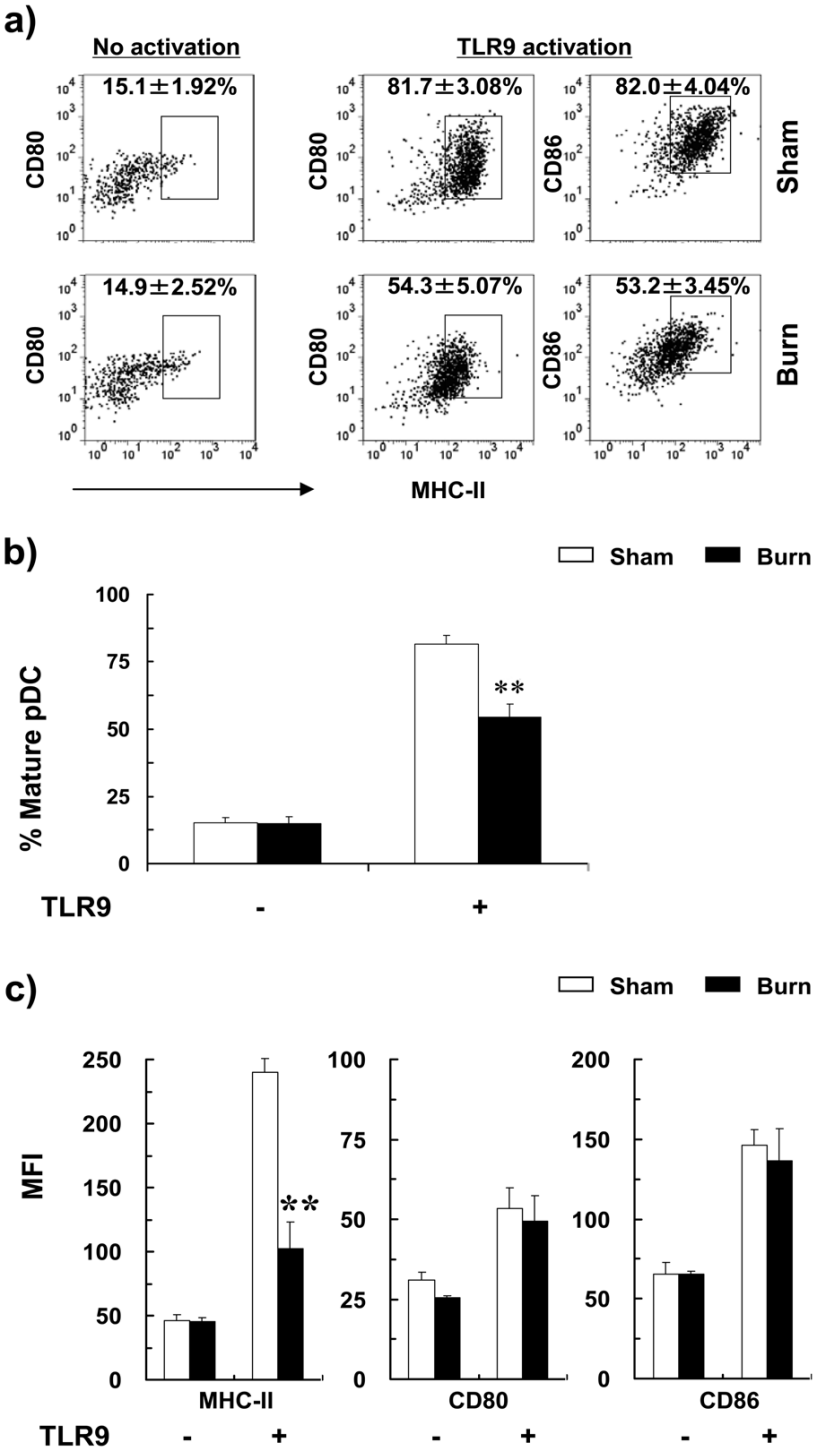
Splenic pDCs of burn-injured mice had a diminished ability to undergo maturation following TLR9 activation. Splenic DCs were enriched on d3 after injury, activated with or without CpG (40 hr) then stained for CD11c, B220, CD80, CD86 and MHC II. PDCs were gated as CD11c^low^B220^+^PDCA1^+^ and the expression of CD80, CD86 and MHC II were analyzed by flow cytometry. (a) Representative FACS plots of non-activated and TLR9-activated pDCs of burn and sham mice. Percentages of mature pDCs (MHCII^hi^CD80^hi^CD86^hi^) are shown as mean ± SEM (n = 9, 3 independent experiments). (b) Percentages of MHCII^hi^CD80^hi^CD86^hi^ mature pDCs and (c) MFI of MHC II, CD80, and CD86 expression on pDCs with and without TLR9 activation are shown as mean ± SEM (n = 9, 3 independent experiments). *P<0.05, **p<0.01, sham versus burn by ANOVA.

### Statistics

Results were analyzed using PRISM version 3.0 software by two-tailed ANOVA. A *p*<0.05 was considered to be significant.

**Figure 6:**
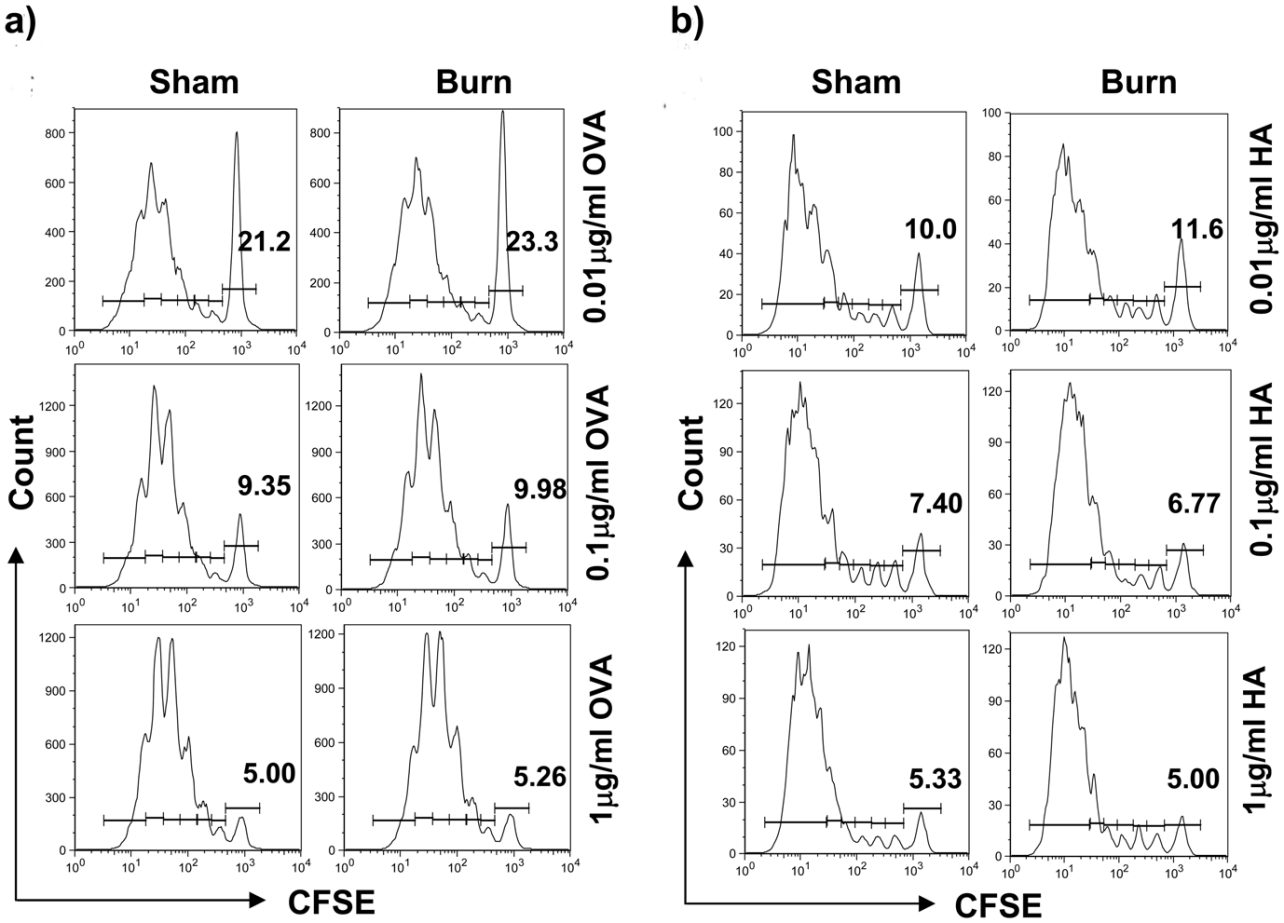
Burn injury had no impact on the ability of cDCs to stimulate Ag-specific T cell proliferation. Three days post-burn/sham injury, FACS-sorted cDCs were pulsed with either OVA_323-229_ class II or HA class I-restricted peptides (concentration ranging from 0.01 to 1 µg/ml) and activated with CpG (6 µg/ml, 18–20 hr). DCs were then washed and subsequently co-cultured with CFSE-labeled CD4^+^ and CD8^+^ T cells for three consecutive days. Proliferation of (a) CD4^+^ and (b) CD8^+^ TCR-transgenic T cells is illustrated by means of CFSE dilution measured using flow cytometry. Representative FACS plots with percentages of unproliferated cells are shown (n = 9, 3 independent experiments).

## Results

### Burn Injury Reduced Splenic cDC and pDC Numbers

To examine the effects of burn injury on DC subsets, mice were subjected to non-lethal thermal injury (25% total body surface, full thickness) and the numbers of cDCs and pDCs at the site of injury, spleen and wound draining lymph nodes (LNs) were examined at various time points thereafter. In the first three days following burn injury, the percentage of splenic CD11c^hi^B220^neg^ cDCs and CD11c^low^B220^+^ DC subpopulations was decreased, comparison to sham-injured animals (Fig. 1a). The CD11c^low^B220^+^ subpopulation consists of pDCs (CD11c^low^B220^+^PDCA1^+^) and IKDCs (CD11c^low^B200^+^DX5^+^) [19], [20], [21]. Staining with specific surface markers further revealed that both the numbers and percentages of splenic cDCs and pDCs decreased for the first three days post-burn injury (Fig. 1b, c). The pDC quantity remained low until day 5 post-injury but returned to normal levels by day 7. As for splenic IKDCs, both the numbers and percentages remained unaltered except a modest decrease at the early time points (Fig. 1*b, c*) [*30*].

**Figure 7:**
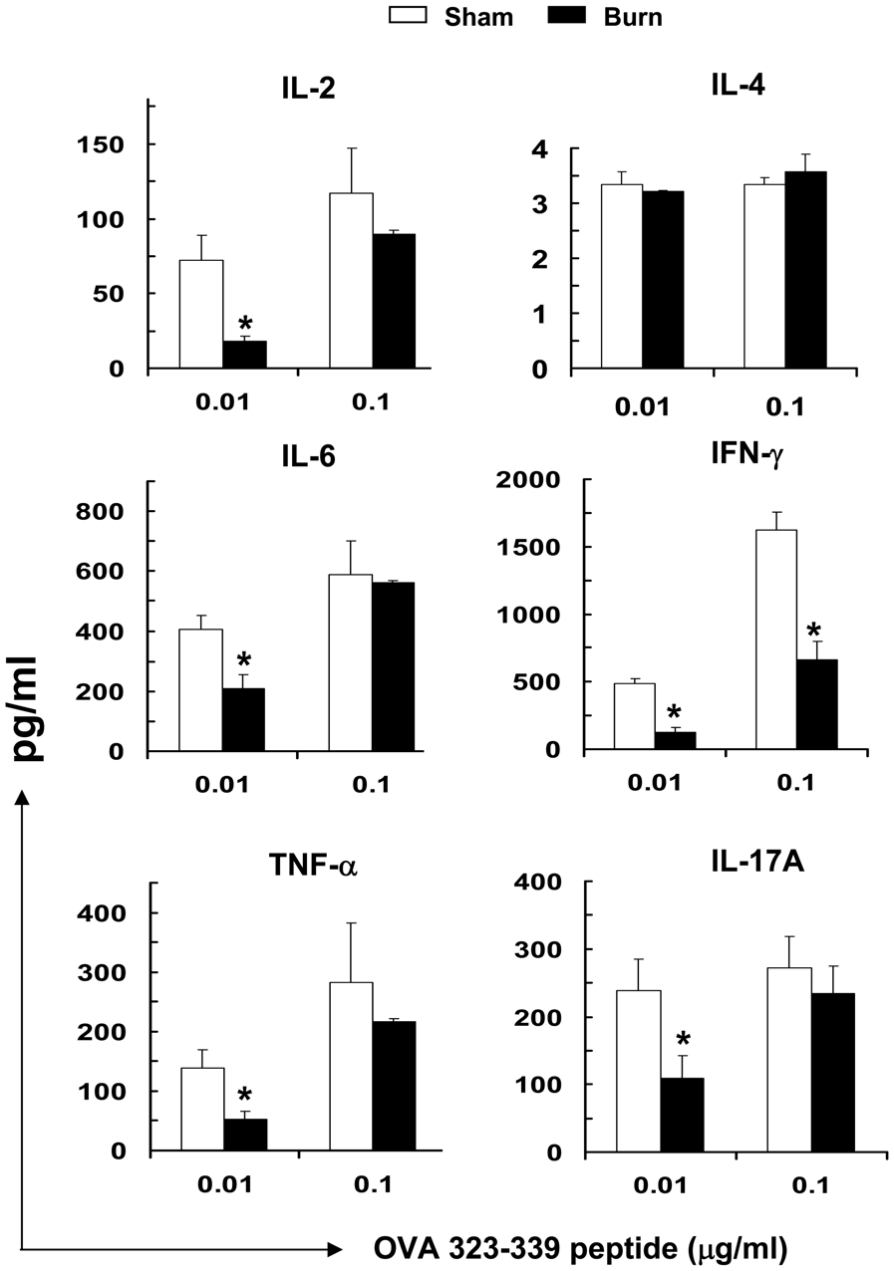
CDCs from burn-injured mice had an impaired ability to trigger Th1 and Th17 CD4^+^ T cell responses. FACS-sorted cDCs were isolated on d3 post injury, pulsed with OVA_323-229_ class II-restricted peptide (0.01 µg/ml to 0.1 µg/ml), then activated with CpG (6 µg/ml, 18–20 hr). CD4^+^ T cells were subsequently co-cultured with the washed, activated cDCs for three days. Cytokines production was measured as pg/ml in supernatants by cytometric bead analysis assay. Data represent mean ± SEM (n = 9, 3 independent experiments). *P<0.05, sham versus burn by ANOVA.

As reflected in Table 1, the absolute numbers and percentages of two CD4^−^CD8α^+^ and CD4^+^CD8α^−^ cDC subpopulations decreased post-burn injury, while the ratio of splenic CD4^+^CD8α^−^ cDCs to CD4^−^CD8α^+^cDCs compared to sham remained unaltered (Table 2). CDC and pDC quantities were also reduced in the wound draining LNs (i.e. two axillary, two brachial, three mesenteric and two inguinal LNs) following burn injury (Table 3) and no significant difference in CD11c^+^ DC infiltration was detected at the injury site between burn and sham groups (Fig. 2). These data suggest that the decreased in splenic DC populations was not due to their migration to the wound draining LNs or the injury site.

**Figure 8:**
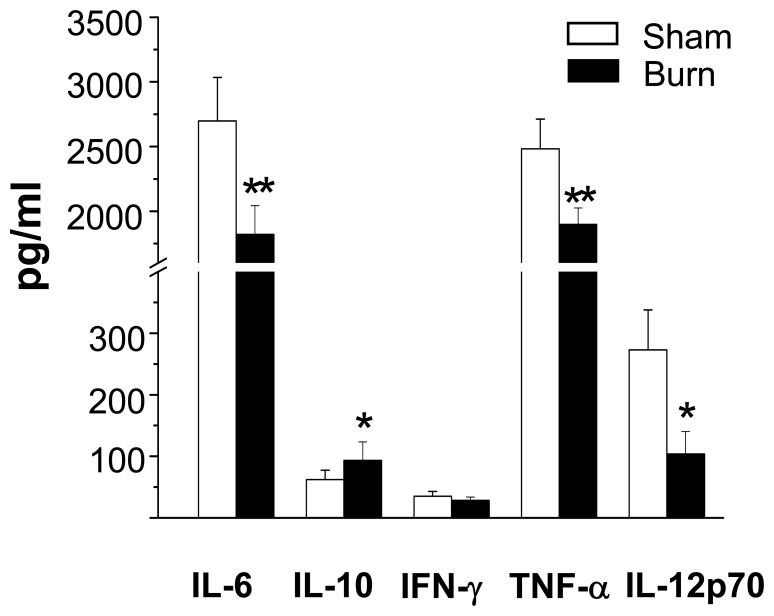
Burn injury impaired splenic cDCs’ ability to secrete pro-inflammatory cytokines upon TLR9 activation. Three days post-injury, FACS-sorted cDCs with purity >98% were activated with CpG (6 µg/ml, 18–20 hr). Pro-inflammatory cytokine production by TLR9-activated cDCs was measured by cytometry bead analysis (CBA) assay. Data represent mean ± SEM (n = 6, 3 independent experiments). *P<0.05; **p<0.01, sham versus burn by ANOVA.

### Burn Injury Impaired TLR9-mediated Maturation of pDCs

We postulate here that burn injury impaired TLR9-induced DC maturation. To this end, we only focused on cDC and pDC populations. To do this, we purified splenic cDCs and pDCs from burn and sham-injured mice at days 1, 3 and 5 post-injury and activated with the TLR9 ligand, CpG. Expression of maturation markers, including MHC II, CD80 and CD86, was measured by FACS. At day 3 post-injury, without TLR activation, cDCs of both burn- and sham-injured mice expressed similar levels of MHC II, CD80 and CD86 (Fig. 3). Upon TLR9 stimulation, the percentage of cDCs that underwent maturation to become mature cDCs (i.e. MHCII^hi^CD80^hi^CD86^hi^) was lower in burn-injured mice compared to sham control (Fig. 3a &b). The expression of MHC II and CD80 were lower in splenic cDCs of burn mice (Fig. 3b &c). Similar responses were observed at days 1, 3 and 5 post-burn injury (Fig. 4a).

**Figure 9:**
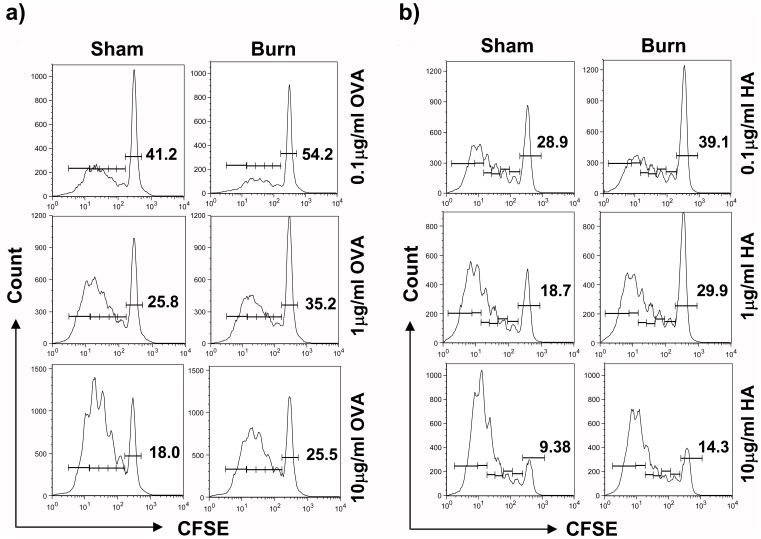
TLR9-activated pDCs from burn-injured mice had a reduced ability to activate CD4^+^ and CD8^+^ T cell proliferation. Three days post-injury, FACS-sorted splenic pDCs were pulsed with either OVA_323-229_ class II or HA class I-restricted peptides (concentration ranging from 0.1 to 10 µg/ml) and activated with CpG (6 µg/ml, 18–20 hr). CFSE-labeled OVA-specific CD4^+^ and HA-specific CD8^+^ T cells were co-cultured with the washed, activated pDCs for three consecutive days. Proliferation of (a) CD4^+^ and (b) CD8^+^ TCR-transgenic T cells is illustrated by means of CFSE dilution measured using flow cytometry. Representative FACS plots are shown and percentages of unproliferated cells were gated (n = 9, 3 independent experiments).

In contrast to the modest effect on splenic cDCs, pDCs of burn-injured mice demonstrated a decreased maturation ability following TLR9 activation (Fig. 5). At day 3 post-injury, the percentage of pDCs that became MHCII^hi^CD80^hi^CD86^hi^ mature pDCs after TLR9 challenge was also lower in burn-injured mice (Fig. 5a & b). The TLR9-activated pDCs from burn-injured mice demonstrated an approximately 2-fold decrease in MHC II expression, measured as the mean fluorescence intensity (MFI) (109.1±20.1 of burn vs. 236.3±10.8 of sham) (Fig. 5c). Similar findings were detected at day 1, 3 and 5 post-burn injury (Fig. 4b).

**Figure 10:**
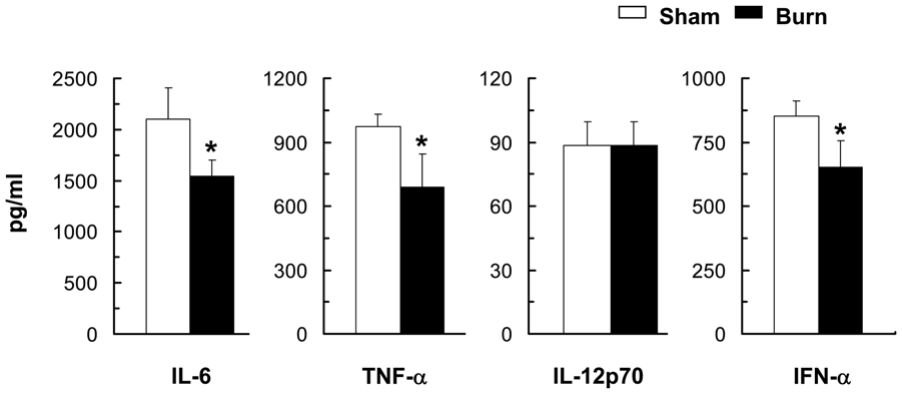
PDCs from burn-injured mice had an impaired ability to secrete pro-inflammatory cytokines. Three days post-injury, FACS-sorted spleen pDCs with purity >98% were activated with CpG (18–20 hr). Inflammatory cytokines and IFN-α productions by TLR9-activated pDCs were measured by CBA assay and ELISA, respectively. Data are shown as mean ± SEM (n = 8, 4 independent experiments). *P<0.05, sham versus burn by ANOVA.

### TLR9-activated-cDCs of Burn-injured Mice had an Aberrant Ability to Drive Th1 and Th17 T Cell Differentiation

To examine whether burn injury affected DCs’ ability to activate and shape T cell responses, splenic TLR9-activated cDCs were pulsed with OVA or HA peptides and co-cultured with OVA-specific CD4^+^ or HA-specific CD8^+^ transgenic T cells, respectively. TLR9-activated cDCs from burn-injured mice did not exhibit an altered ability to induce CD4^+^ and CD8^+^ T cell proliferation (Fig. 6a, b). To assess the ability of cDCs of burn-injured mice on polarizing T helper cell differentiation, we monitored cytokines production by the CD4^+^ T cells activated by splenic TLR9-cDCs. The activated T cells demonstrated aberrant Th1 and Th17 cytokines production, secreting markedly lower levels of Th1 (IFN-γ, IL-2, TNF-α) cytokines and Th17A, compared to T cells that were primed by TLR9-activated cDCs of sham-injured mice (Fig. 7).

**Figure 11:**
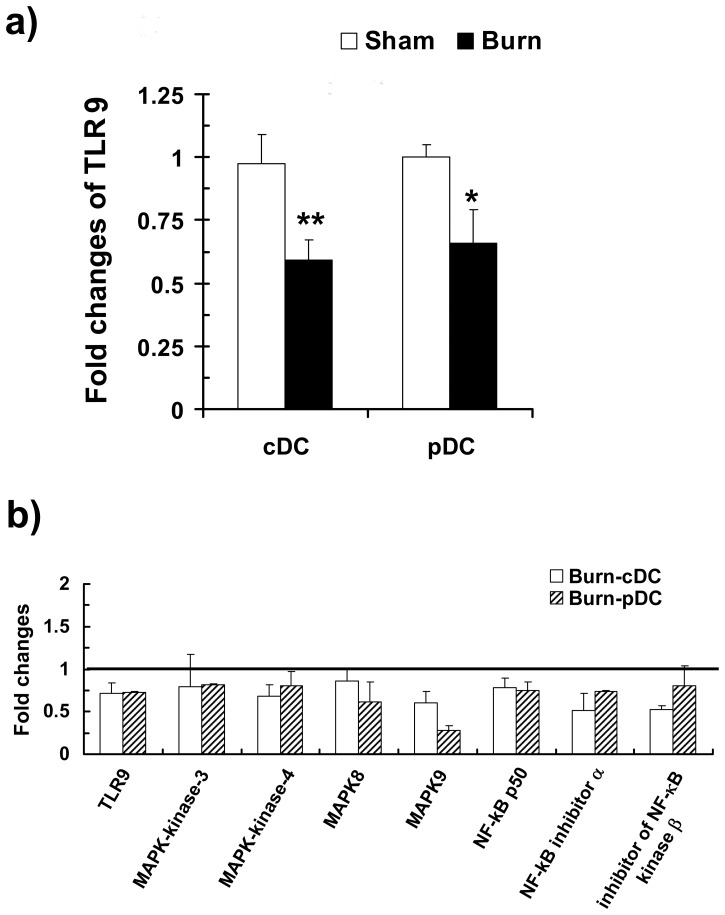
CDCs and pDCs from burn-injured mice expressed a lower transcript level of TLR9 and genes related to TLR signaling pathway. Three days post-injury, cDCs and pDCs were purified by FACS sorting. (a) Transcript level of TLR9 was examined by real-time PCR. Fold changes of TLR expressions of burn and sham mice were normalized to those of untreated control mice. The data analyzed represents the mean ± SEM of 5–6 independent experiments using 10 mice per group. *, *p*<0.05, sham versus burn by ANOVA. (b) The expressions of genes related to TLR-mediated signal transduction were examined by real-time PCR Array. Fold changes of transcript levels of genes in cDCs and pDCs of burn mice were normalized to the corresponding genes of sham mice per experiment. Genes expression in cDC and pDC of sham mice were set as 1. The data analyzed represent the mean ± SEM (n = 10, 2 independent experiments).

### Burn Injury Skewed Cytokines Production Profile of TLR9-activated cDCs

Given TLR9-activated cDCs of burn-injured mice exhibited a reduced ability to polarize Th1 and Th17 T cell response, we next sought to determine whether this could be explained by changes in the profile of cytokine secretion by cDCs themselves. Compared to the sham controls, TLR9-activated cDCs from burn-injured mice showed predominant secretion of the anti-inflammatory cytokine IL-10 (∼50% increase), whereas secretion of the pro-inflammatory cytokines IL-6 (∼30% decrease), TNF-α (∼25% decrease) and IL-12p70 (∼60% decrease) was notably reduced (Fig. 8). To ensure these differences in cytokine production found were not the result of increased cDC death, we evaluated cell viability of TLR9-activated cDCs using Annexin V and propidium iodide. No significant difference in the viability of TLR9-activated cDCs was detected between the burn-injured and sham groups (data not shown).

### PDCs From burn-injured Mice Had an Impaired Ability to Secrete Pro-inflammatory Cytokines and to Induce T Cell Proliferation

In order to determine the biological relevance of pDCs’ impaired maturation in response to TLR9 activation, we next investigated whether pDCs’ ability to induce T cell proliferation was affected. TLR9-activated pDCs of burn-injured mice exhibited a functional impairment, with an aberrant ability to trigger antigen-specific CD4^+^ and CD8^+^ T cell proliferation (Fig. 9). This impairment was accompanied by a reduced secretion of IFN-α, IL-6 and TNF-α compared to sham controls (Fig. 10).

### Burn Injury Reduced DCs’ Transcript Abundance of TLR9 and Other Molecules in the TLR Signaling Pathway

To glean insights into the mechanism responsible for the altered DC responses to TLR9 activation, we performed a gene expression profiling of the TLR signaling pathway in cDCs and pDCs of burn- and sham-injured mice. We found that cDCs and pDCs from burn-injured mice expressed lower TLR9 transcript levels compared to the sham controls (Fig. 11a). Using TLR signaling pathway-specific qRT-PCR arrays, we observed that the expression of all of the studied TLR signaling molecules were down-regulated in cDCs and pDCs of burn-injured mice, compared to those of sham (Fig. 11b).

## Discussion

Severe injury promotes an imbalance of innate and adaptive immune responses, resulting in compromised host defenses and increased susceptibility to infection. Evidence suggests that, following burn injury, the innate immune cells acquire an exacerbated TLR reactivity that has been attributed to SIRS [1], [2]. In the present study, we examined the effect of burn injury on the responsiveness of two major DC populations, cDCs and pDCs, to TLR9 activation. Our findings show that burn injury reduced the splenic cDC and pDC populations and altered their TLR9 reactivity. Mouse splenic cDCs are broadly classified into two major subsets, each with distinct functions: CD4^−^CD8α^+^cDCs, which express DEC-205, efficiently cross-present to CD8^+^ T cells [30] and produce bioactive IL-12p70, a cytokine involved in inducing Th1 cell responses; CD4^+^CD8α^−^ cDCs, by contrast, efficiently produce pro-inflammatory chemokines. Splenic CD4^−^CD8α^+^ and CD4^+^CD8α^−^ cDC subpopulations were decreased upon burn injury. Burn injury altered the ability of cDCs to secrete pro-inflammatory cytokines in response to TLR9 activation; thus, impaired their ability to effectively prime Th1 and Th17 T cell responses. Following burn injury, pDCs also demonstrated altered immunobiology, with a reduced ability to activate antigen-specific proliferation of CD4^+^ and CD8^+^ T cells and to secrete pro-inflammatory cytokines.

DCs recognize microbes via PRRs, including the TLR family, and therefore play a central role in activating host defenses against microbes. However, the role of DCs in injury-induced immune dysregulation remains unclear. Fujimi *et al*. reported that severe injury does not negatively affect the antigen-presenting function of cDCs but does reduce their TLR4 reactivity [31]. Other investigators have found that severe injury triggers DC dysfunction, which results in immune suppression. For example, Patenaude *et al.* demonstrated that burn injury down-regulates TLR4/MD-2 expression on splenic CD11c^+^CD8α^+^ cDCs and disrupts their TLR4 reactivity [32]. Kawasaki *et al.* showed that severe injury attenuates the production of TNF-α, IL-6 and IFN-γ by cDCs [33]. Burn injury has also been associated with decreased TLR4 expression in dermal DCs as well as altered function of epidermal Langerhans cells [34], [35], [36]. Furthermore, fms-like tyrosine kinase 3 ligand treatment after burn injury promotes resistance to wound infection by augmenting neutrophil function in a DC-dependent manner [37].

Our findings support the role of DCs in injury-induced immune dysfunction. We demonstrate abnormal responses of cDCs and pDCs to TLR9 activation following burn injury. The release of important pro-inflammatory cytokines, including IL-12, TNF-α and IL-6 and IFN-α, required for productive innate and adaptive immune functions, was compromised in both cDCs and pDCs. Production of these cytokines is essential for functional differentiation and commitment of helper T cells to Th1 and Th17 phenotypes. Th1 and Th17 T cells are important for the clearance of pathogens such as *Staphylococcus aureus, Pseudomonas aeruginosa,* and the fugus *Candida albicans* [38], [39]. Hence, an impaired repertoire of pro-inflammatory cytokines during infection can have serious negative consequences on the infected host. For instance, IL-12 release is critical for the elimination of intracellular bacteria and viruses, as it is a potent activator of NK cells [40], NKT cells [41] neutrophils [42], and plays an important role in Th1 differentiation. IL-6 and TNF-α are also important for Th17 differentiation and anti-microbial responses [43]. Our findings here suggest that cDCs are unlikely contribute to the injury-induced SIRS; rather, they promote an anti-inflammatory environment. At low peptide concentrations, cDCs of burn-injured mice had a reduced ability to trigger Th1 and Th17 T cell responses. This suggests that cDCs’ functional impairment may be particularly aggravated in early infection stages when the abundance of pathogenic antigens is low. Signaling through TLRs enables DCs to promote a rapid and effective response against invading pathogens [25], [27], [28]. TLR9 activation triggers signal transduction pathways that result in the activation of MAPK, NF-κB, and the IFN regulatory factor (IRF) family. Alterations in the expression of these factors can cause defects in DC maturation as well as cytokine and chemokine production. Our gene-expression array data revealed that the abundance of both TLR9 transcripts and of many key TLR signaling molecules were reduced in cDCs and pDCs following burn injury. Though the mechanism by which TLR9 is down-regulated post-burn-trauma awaits clarification, the down-regulation of TLR9 and its signaling pathway may be responsible for the observed defects in DC functions detected in this study. We are, at present, investigating the mechanism underlying how TLR9 signaling is affected in cDCs and pDCs, as well as whether signaling of other TLRs is affected.

TLR9 plays a role in bacterial DNA recognition [44] and its activation is required for an effective host anti-bacterial immune response [45], [46], [47]. Although TLR9 is found in cDCs and pDCs in mice, it is exclusively expressed in pDCs in humans. Thus, pDCs are considered a major effector of antiviral immunity in humans. To date, studies on the TLR reactivity of DCs after burn injury have largely focused only on CD11c^hi^ DCs; only a limited cohort of studies have investigated functional characteristics of pDCs in burn injury. In addition to playing different roles in shaping the innate and adaptive immune responses, cDCs and pDCs are engaged in crosstalk with each other to enhance immunological outcomes [48], [49]. Thus, functional impairment of one DC population can deleteriously affects the other’s function. Recent investigations on the biology of different DC subsets confirm the importance of inter-DC interactions. Here, we demonstrated that, in addition to an inefficient production of pro-inflammatory cytokines, pDCs failed to trigger effective CD4^+^ and CD8^+^ T cell proliferation. To our knowledge, this is the first study that reported a functional impairment of pDCs following severe injury. Future studies to further elucidate the cellular and molecular events that result in DCs’ dysfunctions and how these contribute to the induced immunosuppression oftenly observed post-injury are needed. Such insight, in conjunction with that contributed by the present study, will be critical for the development of therapies to enhance immunity and decrease morbidity following devastating burn injuries.
